# *SAA1* gene polymorphisms in osteoporosis patients

**DOI:** 10.1042/BSR20181031

**Published:** 2019-02-19

**Authors:** Xindie Zhou, Jin Li, Lifeng Jiang, Dong Zhou, Lidong Wu, Yong Huang, Nanwei Xu

**Affiliations:** 1 Department of Orthopedics, The Affiliated Changzhou No. 2 People’s Hospital of Nanjing Medical University, Changzhou 213003, China; 2 Department of Orthopedics Surgery, The Second Affiliated Hospital of Jiaxing University, Jiaxing 31400, China; 3 Department of Orthopedics Surgery, The Second Affiliated Hospital, Zhejiang University School of Medicine, Hangzhou 310000, China

**Keywords:** Chinese population, molecular epidemiology, osteoporosis, polymorphism, SAA1

## Abstract

**Background:** Serum amyloid A (SAA1) is an apolipoprotein that maintains glucose and lipid homeostasis. Its polymorphisms are associated with risks of myocardial infarction and coronary artery disease (CAD). **Methods:** However, little is known about the associations of these polymorphisms with susceptibility to osteoporosis, which we evaluated in this hospital-based case–control study involving 300 osteoporosis patients and 350 controls. Three single-nucleotide polymorphisms (SNPs) (rs183978373, rs12218, and rs10832915) were genotyped using MALDI TOF MS. **Results:** There were no differences in the rs183978373 and rs12218 polymorphisms between the osteoporosis group and controls. The *SAA1* gene rs10832915 polymorphism increased the risk of osteoporosis in our Chinese population. The genotypes of the rs10832915 polymorphism were not significantly associated with clinical parameters (age, body mass index (BMI), high- and low-density lipoprotein (LDL), total cholesterol (TC), and T-score). Haplotype analysis revealed that the ATT haplotype had a significant correlation with a decreased risk of osteoporosis. **Conclusion:** In conclusion, the *SAA1* rs10832915 polymorphism and its haplotypes are associated with osteoporosis, but this finding should be confirmed in large well-designed studies.

## Introduction

Osteoporosis is the most common bone disease and is characterized by a reduction in bone mineral density (BMD), a deterioration of the bone tissue microarchitecture, and an increased risk of fractures [1]. The prevalence of osteoporosis is higher in females than in males (25.41 compared with 15.33%) and increases with age [2]. Despite its severe effects, the mechanism of underpinning osteoporosis remains unclear. Osteoporosis is recognized as a multifactorial disease resulting from genetic, and environmental factors and their interaction [3,4]. A genetic study revealed that osteoporosis has a major risk of low BMD and is controlled genetically [5].

Serum amyloid A (SAA) proteins are a family of inflammatory apolipoproteins that may modify the structures and functions of high-density lipoprotein (HDL) [6]. The higher HDL-C level of high-SAA1 patients is associated with increased all-cause and cardiovascular mortality rates [7]. SAA1 plays an active role in atherosclerosis and coronary artery disease (CAD) [6,8]. The incidence of CAD including a high coronary artery calcium score (≥100), obstructive CAD, and multivessel disease is significantly higher in lower BMD subjects, including those with osteopenia and osteoporosis [9]. However, no association between SAAs and osteoporosis has been reported.

*SAA1* is located in chromosome 11p15.1 and has four exons. Although two studies have reported an association between the *SAA1* rs12218 polymorphism and the risk of osteoporosis, their findings conflicted [10,11]. Therefore, this case–control study which enrolled 300 osteoporosis patients and 350 controls was conducted to evaluate the role of *SAA1* gene polymorphisms in the risk of osteoporosis in a Chinese population.

## Materials and methods

### Participants

The 300 postmenopausal osteoporosis patients and 350 controls were enrolled from the Affiliated Changzhou No. 2 People’s Hospital of Nanjing Medical University (Changzhou, China) and the Second Affiliated Hospital of Medical College, Zhejiang University (Hangzhou, China) with a respond rate of 78% between April 2012 and June 2017. All participants were of Chinese Han nationality and genetically unrelated. Those having diseases (e.g. diabetes mellitus, hyperthyroidism, or any systemic illness) or taking drugs (e.g. calcium supplements) that affected bone metabolism were excluded. The present study was approved by the Institutional Ethnics Committees of the two hospitals. Personal and medical confidentiality was ensured in line with Declaration of Helsinki. Written informed consent was obtained from all participants.

### Measurement of body mass density

BMDs at the lumbar spine (L2–L4) and the femoral neck were measured using a dual-energy X-ray absorptiometer (Lunar Corp, Madison, WI, U.S.A.) by two qualified radiologists who were blind to other medical data. BMDs were recorded in g/cm^2^ and as peak bone mass percentage in the controls (T-score).

### Co-expression analysis and single-nucleotide polymorphism selection

The co-expressed genes with SAA1 were identified on MEM-Multi Experiment Matrix (http://biit.cs.ut.ee/mem/index.cgi) Relevant functions of these genes were explored through Gene Ontology (GO) analysis.

Linkage data of *SAA1* gene were searched from Ensembl (http://www.ensembl.org/index.html) and processed on Haploview. Relevant parameters were set as follows: Hardy–Weinberg *P*-value cutoff = 0.05; minimum genotype = 95%; Max# Mendel error = 1; minimum allele frequency = 0.05. Such setting may help to find the tagging single-nucleotide polymorphisms (SNPs).

### Peripheral blood DNA extraction and genotyping for *SAA1* gene

Peripheral venous blood (2 ml) was collected from each participant, and then genomic DNA was isolated using a WizardVR genomic DNA purification kit (Promega, Madison, U.S.A.) following the supplier’s manual. The concentration and purity of the genomic DNA were estimated on NanoDrop using two optical density wavelengths 260 and 280 nm.

Genotyping was done by MALDI-TOF-MS using a MassARRAY system (Sequenom, San Diego, CA, U.S.A.) as previously described [13]. Complete genotyping reactions were spotted on to a 384-well spectroCHIP (Sequenom) using MassARRAY nanodispenser (Sequenom) and analyzed by MALDI-TOF-MS. Genotype callings were done in real time with MassARRAY RT 3.1 and analyzed on MassARRAY Typer 4.0 (both Sequenom). For quality control, 10% of the randomly selected samples were analyzed repeatedly.

### Genotype and gene expression correlation analysis

Genotypes data of *SAA1* rs10832915 polymorphism were downloaded from the International HapMap Project. Its mRNA expression data were available on GTex portal (https://www.gtexportal.org/home/) [12].

### Statistical analysis

All statistical analyses were conducted on Stata 11.0 (SAS Institute, Cary, NC, U.S.A.). All continuous variables were expressed as mean ± S.D. Hardy–Weinberg equilibrium (HWE) for the rs12218 polymorphism in the control group was examined using the Chi-square test. Differences in BMDs, body mass index (BMI), HDL, low-density lipoprotein (LDL), total cholesterol (TC), T-score and Z-score according to different genotypes were tested using analysis of covariance (ANOVA). Association between variables was evaluated by multiple regression and logistic regression. Odds ratio (OR) and 95% confidence interval (CI) were calculated to evaluate the association between the *SAA1* rs12218 polymorphism and the risk of osteoporosis by logistic analysis. The significance was set at *P*<0.05.

## Results

### Bioinformatics analysis

**Figure 1:**
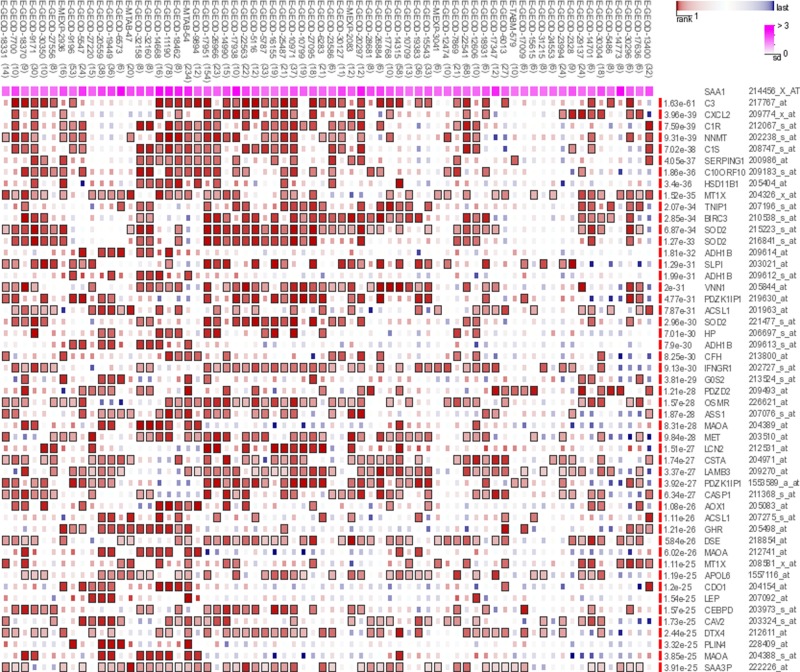
Co-expression genes with SAA1

**Figure 2:**
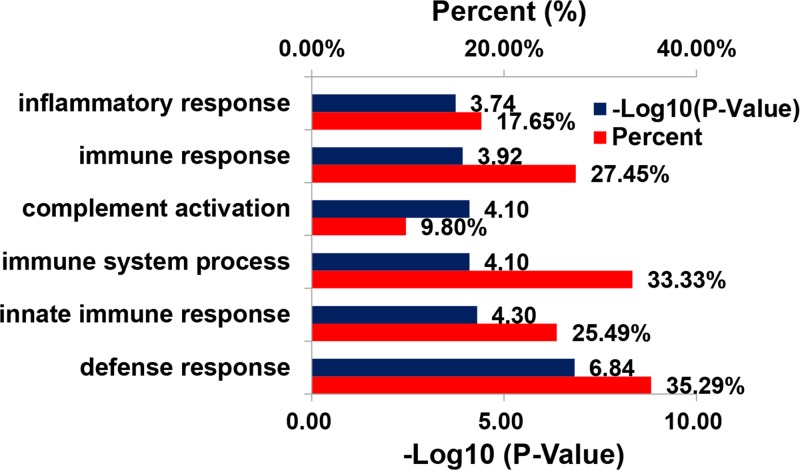
Barplot of representative GO biological process of co-expressed gene with SAA1

The genes co-expressed with *SAA1* are shown in Figure 1. The cortisol synthesis enzyme 11β-hydroxysteroid dehydrogenase (HSD11B) affects the physiological levels and severity of age-related osteoporosis [13]. Superoxide dismutase 2 (SOD2) was significantly up-regulated at protein level *in vivo* in low-hip-BMD Chinese compared with high-hip-BMD [14]. GO analysis of biological process indicated that these genes of *SAA1* were mainly involved in the immune system process (Figure 2).

**Figure 3:**
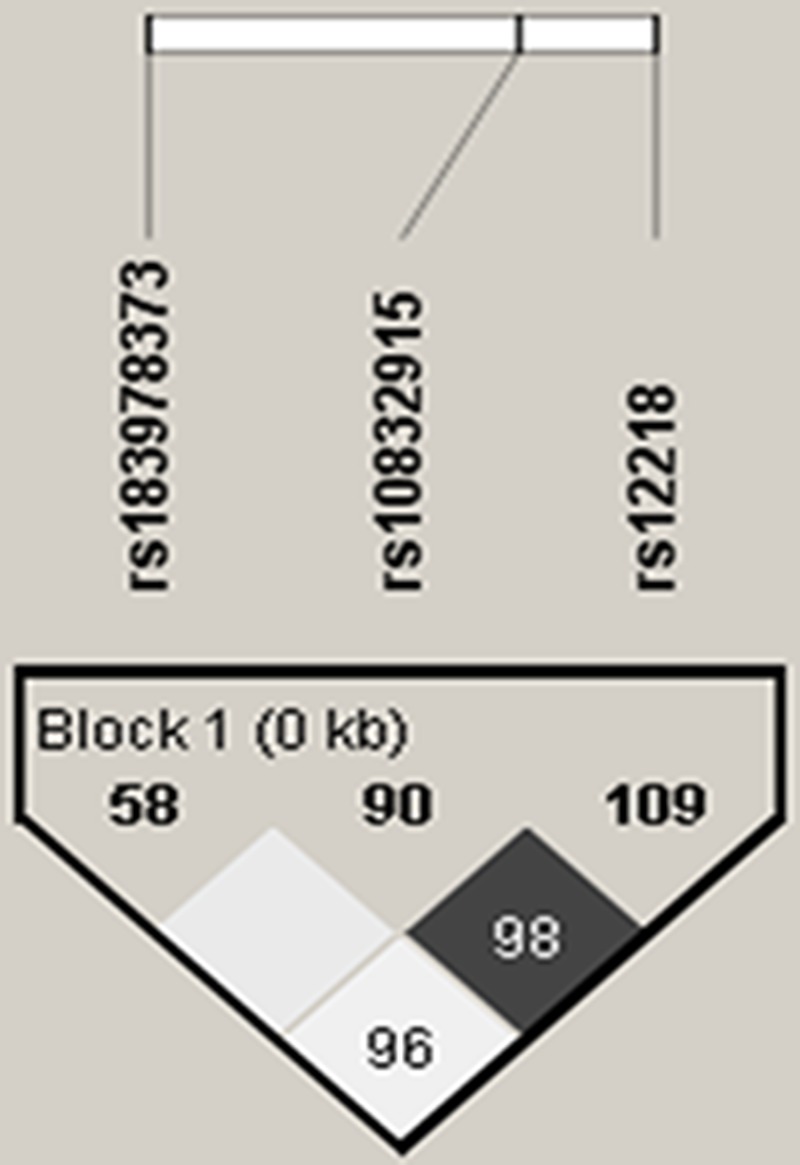
Linkage disequilibrium for *SAA1* gene

In the present study, three tagging SNPs (rs183978373, rs12218, and rs1083295) were identified. Of them, rs12218 was under linkage disequilibrium with two other SNPs that were also located in the intron region of *SAA1* gene (Figure 3).

### Subject characteristics

Some characteristics of the study population and corresponding significances were summarized in Table 1. The mean ages were not significantly different between the patients and the controls (46.70 compared with 46.88 years). Significant differences between the cases and the controls were found in T-scores and Z-scores (both *P*<0.05), but not in BMI, HDL, LDL, or TC (all *P*>0.05). No significant deviations of the SAA1 gene polymorphisms from HWE were found in either patients or controls (*P*=0.658, 0.975, and 0.856, respectively).

**Table 1:** Patient demographics and risk factors in osteoporosis

Variable	Cases (*n*=300)	Controls (*n*=350)	*P*
Age (years)	46.70 ± 4.47	46.88 ± 5.01	0.641
BMI (kg/m^2^)	24.40 ± 1.45	24.24 ± 1.45	0.179
HDL (mmol/l)	1.21 ± 0.35	1.24 ± 0.36	0.241
LDL (mmol/l)	2.61 ± 0.84	2.43 ± 0.75	0.050
TC (mmol/l)	4.48 ± 0.96	4.38 ± 0.84	0.143
T-score	−3.02 ± 0.58	0.13 ± 0.11	<0.001
Z-score	−2.08 ± 0.47	0.63 ± 0.18	<0.001

### Analysis of *SAA1* gene polymorphisms

**Figure 4:**
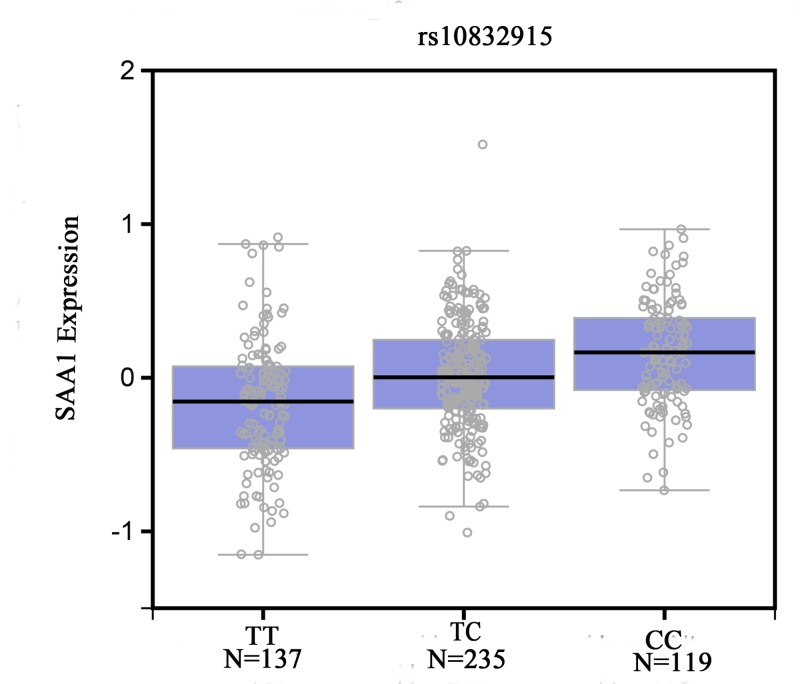
Functional implication of SAA1 rs10832915 polymorphism

The genotype and allele distributions of the three *SAA1* gene polymorphisms in the two groups are provided in Table 2. *SAA1* rs183978373 polymorphism was not associated with the risk of osteoporosis. The rs12218 polymorphism was significantly correlated with an increased risk of osteoporosis in the recessive model. The CC genotype or C allele of rs10832915 polymorphism was significantly associated with the risk of osteoporosis (CC compared with TT, OR = 1.62, 95% CI = 1.01–2.60, *P*=0.045; C compared with T, OR = 1.26, 95% CI = 1.01–1.58, *P*=0.041, Table 2). No association between this polymorphism and risk of osteoporosis was identified under the dominant or recessive models. The genotypes of this polymorphism were not significantly associated with clinical characteristics (age, BMI, TC, HDL, LDL, and T-score; Table 3). The *SAA1* mRNA expression levels by the genotypes of rs10832915 polymorphism were shown in Figure 4. We found significant difference in the expression levels for rs10832915 polymorphism in the muscles (*P*=1.7 × 10^−12^). CC genotype increased the SAA1 compared with TT genotype. Furthermore, all three SNPs were located in one haplotype block. Haplotype analysis suggested ATT haplotype was associated with decreased risk of osteoporosis (Table 4).

**Table 2:** Logistic regression analysis of associations between SAA1 gene polymorphisms and risk of osteoporosis

Genotype	Cases (*n*=300)		Controls (*n*=350)		OR (95% CI)	*P*
	*n*	%	*n*	%		
Rs183978373						
CC	225	75.0	245	70.0	1.00	
CA	65	21.7	97	27.7	0.73 (0.51, 1.05)	0.088
AA	10	3.3	8	2.3	1.36 (0.53, 3.51)	0.524
AA+CA	75	25.0	105	30.0	0.78 (0.55, 1.10)	0.156
CC+CA	290	96.7	342	97.7	1.00	
AA	10	3.3	8	2.3	1.47 (0.57, 3.78)	0.420
C allele	515	85.8	587	83.9	1.00	
A allele	85	14.2	113	16.1	0.86 (0.63, 1.16)	0.323
Rs12218						
TT	98	32.7	113	32.3	1.00	
TC	137	45.7	187	53.4	0.85 (0.60–1.20)	0.343
CC	65	21.7	50	14.3	1.50 (0.95–2.37)	0.083
TC+CC	202	67.3	237	67.7	0.98 (0.70–1.36)	0.918
TT+TC	235	78.4	300	85.7	1.00	
CC	65	21.7	50	14.3	**1.66 (1.11–2.49)**	0.015
T allele	333	44.5	413	59.0	1.00	
C allele	267	55.4	287	41.0	1.15 (0.93–1.44)	0.203
Rs10832915						
TT	108	36.0	148	42.3	1.00	
TC	140	46.7	158	45.1	1.21 (0.87, 1.70)	0.258
CC	52	17.3	44	12.6	**1.62 (1.01, 2.60)**	0.045
TC+CC	192	64.0	202	57.7	1.30 (0.95, 1.79)	0.102
TT+TC	248	82.7	306	87.4	1.00	
CC	52	17.3	44	12.6	1.46 (0.94, 2.25)	0.089
T allele	356	40.7	554	64.9	1.00	
C allele	244	59.3	246	35.1	**1.26 (1.01, 1.58)**	0.041

Bold values are statistically significant (*P*<0.05).

**Table 3:** The clinical and biochemical characteristics of SAA1 rs10832915 polymorphism between two groups

	Patients (*n*=300)				Controls (*n*=350)			
	TT (*n*=108)	TC (*n*=140)	CC (*n*=52)	*P*	TT (*n*=148)	TC (*n*=158)	CC (*n*=44)	*P*
Age (years)	46.74 ± 4.46	46.58 ± 4.35	46.96 ± 4.88	0.866	46.57 ± 5.03	47.27 ± 5.06	46.50 ± 4.76	0.408
BMI (kg/m^2^)	24.45 ± 1.41	24.47 ± 1.49	24.12 ± 1.40	0.283	24.30 ± 1.45	24.29 ± 1.62	23.91± 1.31	0.310
TC (mmol/l)	4.48 ± 0.94	4.52 ± 0.98	4.41 ± 0.96	0.794	4.30 ± 0.85	4.43 ± 0.86	4.46 ± 0.74	0.329
HDL (mmol/l)	1.22 ± 0.37	1.19 ± 0.34	1.21 ± 0.38	0.730	1.22 ± 0.35	1.25 ± 0.35	1.27 ± 0.43	0.676
LDL (mmol/l)	2.68 ± 0.80	2.61 ± 0.84	2.46 ± 0.90	0.293	2.42 ± 0.70	2.50 ± 0.78	2.25 ± 0.73	0.148
T-score	−3.09 ± 0.54	−3.00 ± 0.61	−2.95 ± 0.57	0.255	0.14 ± 0.11	0.14 ± 0.11	0.13 ± 0.10	0.877
Z-score	−2.11 ± 0.41	-2.06 ± 0.47	−2.11 ± 0.55	0.615	0.62 ± 0.17	0.66 ± 0.19	0.60 ± 0.17	0.094

**Table 4:** Distribution of haplotypes

Haplotype	Osteoporosis	Controls	OR (95% CI)*	*P*
CTT	0.310	0.366	0.85 (0.66, 1.08)	0.184
CCC	0.202	0.193	1.05 (0.78, 1.40)	0.763
ATT	0.077	0.116	**0.66 (0.45, 0.98)**	0.039
CCT	0.273	0.286	0.96 (0.74, 1.24)	0.736

Bold values are statistically significant (*P*<0.05).

* OR, the ratio of the number of individuals with/without CTT/CCC/ATT/CCT haplotype in the osteoporosis patients/the ratio of the number of individuals with/without CTT/CCC/ATT/CCT haplotype in the controls.

## Discussion

We found that *SAA1* rs10832915 polymorphisms conferred susceptibility to osteoporosis under codominant and allelic models. Furthermore, the ATT haplotype decreased the risk of osteoporosis.

During the acute-phase response, SAA1 can remodel HDL in a manner that releases apoA-1 or other efficient ABCA ligands from HDL [15]. An elevated HDL level was observed in osteoporosis patients, and HDL was inversely correlated with BMD [16]. Additionally, SAA induces monocyte tissue factor, which may contribute to inflammation-associated thrombosis [17]. Sun et al. [18] found that SAA alters macrophage phenotypes and modulates macrophage functions via an MYD88-dependent mechanism that affects the magnitude and duration of the inflammatory response. Inflammation modulates bone resorption via proinflammation cytokines and macrophage colony stimulating factor [19]. However, there are few reports on the role of SAA1 in the etiology of osteoporosis.

*SAA1* gene polymorphisms may alter the gene expression and function and modulate osteoporosis susceptibility. Therefore, we performed this case–control study to evaluate the association between *SAA1* gene polymorphisms and the risk of osteoporosis. This is the first study to uncover an association between rs183978373 and rs10832915 polymorphisms and the risk of osteoporosis. The rs12218 polymorphism is associated with the risks of many diseases, including myocardial infarction [20], CAD [21], and ischemic stroke [22]. However, little is known about its association with the susceptibility to osteoporosis. The first relevant hospital-based study involving 307 osteoporosis patients and 387 controls [10]. They found that the CC genotype of the rs12218 polymorphism expressed more SAA1 and increased the risk of osteoporosis compared with the TT genotype [10]. This SNP was also associated with the TC, HDL, LDL levels, and the BMD in osteoporosis patients [10]. Moreover, CC genotype carriers had a lower risk of osteoporosis than TT genotype carriers in a Saudi population including 138 osteoporosis patients and 128 controls [11]. The CC genotype and C allele were associated with TC, LDL, HDL, T-score, Z-score and lower SAA1 levels in osteoporosis patients [11]. Our results suggest that the *SAA1* rs12218 polymorphism is not associated with the risk of osteoporosis in a Chinese Han population involving 300 cases and 350 controls. Additionally, the CC genotype could not significantly change the SAA1 expression; this result stands in contrast with that reported by Feng et al. [10] study who found and increase, as well as to that reported by Abdu-Allah et al. [11], who found a decrease. We failed to find any significant association between the C allele and other clinical parameters (age, BMI, TC, LDL, HDL, and T-score). The disparities between previous studies and ours may be attributed to clinical heterogeneity, the use of different ethnic populations, and small sample sizes. Specifically, the C-allele frequencies of Asians were 0.189 [10] and 0.383 (the present study), which were significantly lower than that in Caucasians (0.734 [11]). This result may be reflected by the difference in the allele frequency of the *SAA1* rs12218 polymorphism. Addtionally, the relatively small sample size may have underpowered the study performed by Abdu-Allah et al. [11]. Finally, the contradictions between our study and Feng et al. [10] may also be explained by the fact that environmental factors (e.g., geographic location and eating habits) also affect susceptibility to osteoporosis.

Three aspects of our results warrant explanation. For rs12218, the amino acid does not change when the nucleotide T is replaced with C. In general, this SNP does not contribute to the risk of osteoporosis. Additionally, the sites linked to the rs12218 polymorphism were found to be located in the intron region of the *SAA1* gene and did not affect the binding of transcription factors or miRNAs to the *SAA1* gene. For rs10832915, the CC genotype was associated with elevated levels of SAA1. We hypothesized that this polymorphism conferred susceptibility to osteoporosis by regulating the SAA1 level.

The present study has several potential limitations. First, selection bias may exist because all participants were from the same hospitals. Second, only three polymorphisms of the *SAA1* gene were examined, and this did not completely cover the gene. Third, our results were affected by confounding factors such as drinking, smoking histories, and occupation. Finally, the sample size was moderate, which could lead to deviation from the true results.

In conclusion, the *SAA1* rs10832915 polymorphism may be a genetic contributor to the risk of osteoporosis in the Chinese Han population. Nevertheless, this finding should be validated by further studies of larger populations and a functional evaluation of the polymorphisms.

## Abbreviations

BMD: bone mineral density
BMI: body mass index
CAD: coronary artery disease
CI: confidence interval
GO: gene ontology
HDL: high-density lipoprotein
HWE: Hardy–Weinberg equilibrium
LDL: low-density lipoprotein
OR: odds ratio
SAA1: serum amyloid A
SNP: single-nucleotide polymorphism
TC: total cholesterol
